# The Bruxoff Device as a Screening Method for Sleep Bruxism in Dental Practice

**DOI:** 10.3390/jcm8070930

**Published:** 2019-06-28

**Authors:** Klara Saczuk, Barbara Lapinska, Paulina Wilmont, Lukasz Pawlak, Monika Lukomska-Szymanska

**Affiliations:** Department of General Dentistry, Medical University of Lodz, 251 Pomorska St., 92-213 Lodz, Poland

**Keywords:** sleep bruxism, bruxism screening, Bruxoff device

## Abstract

Sleep bruxism (SB) is a masticatory muscle activity during sleep and a common phenomenon. Severe SB can have a serious impact on the success of dental treatment. Reliable methods of screening and diagnosing patients with SB are crucial. Therefore, in this study, a Bruxoff device as a potential screening and diagnostic method for sleep bruxism was evaluated. A total of 60 adults participated in this study: 35 patients with symptoms of bruxism (study group) and 25 asymptomatic patients (control group). Data were recorded using Bruxoff. All symptomatic patients participating in the study turned out to be bruxers, while not all asymptomatic patients turned out to be non-bruxers according to the Bruxoff device. Bruxoff is a simple screening device that can be safely used to evaluate masseter muscle activity during sleep. Since the device does not have a built-in microphone and/or video camera and, therefore, cannot record grinding sounds, the objective capabilities of Bruxoff as a single diagnostic device for sleep bruxism are limited.

## 1. Introduction

### 1.1. Definition of Bruxism

For years, due to its multifaceted aetiology, complex implications and ever-changing definitions, bruxism and its diagnosis have remained quite a conundrum despite being a common phenomenon. An estimated prevalence of bruxism ranges from 8% to 31% in the adult population [1], and some research states that bruxism affects women more commonly than men, due to women being more sensitive to stress and reporting it more often [2,3,4]. In accordance with the American Academy of Sleep Medicine (AASM), bruxism is defined as a “repetitive jaw-muscle activity characterized by clenching or grinding of the teeth and/or by bracing or thrusting of the mandible” [5,6].

The complexity of the aetiology of bruxism, however, has caused researchers to go into further study. The previous, commonly used and applied, definition of bruxism from 2013 [7] has been recently revised and recommended to be “retired” in favour of two differentiating definitions published in the widely recognized 2018 International Consensus on the Assessment of Bruxism [8]:

1. Sleep bruxism (SB) is a masticatory muscle activity during sleep that is characterized as rhythmic (phasic) or non-rhythmic (tonic) and is not a movement disorder or a sleep disorder in otherwise healthy individuals.
2. Awake bruxism is a masticatory muscle activity during wakefulness that is characterized by repetitive or sustained tooth contact and/or by bracing or thrusting of the mandible and is not a movement disorder in otherwise healthy individuals.

### 1.2. Aetiology of Bruxism

As seen in the 2018 definition, bruxism is said to have two separate circadian manifestations. In terms of sleep bruxism, it has been said to occur not only during long periods of sleep, but also periods as short as a nap [9,10].

In earlier years, theories linking bruxism with malocclusion and anatomical anomalies gained popularity [11,12]. However, most experts in the field state that there is no such relationship [13,14]. Due to the insufficient amount of well-designed clinical studies, a connection between bruxism and local or peripheral malfunctions or oral health disorders is poorly investigated and documented [15,16,17,18]. Strictly occlusal therapies in bruxist patients with full and healthy dentition have not been heavily supported by experimental evidence [19].

The latest and most recognized theories describe a significant connection between rhythmic (phasic) masticatory muscle activity (RMMA) observed in sleep bruxism and an increased activity from the central nervous system. It has been shown that most SB episodes were observed in relation to both brain and brief cardiac reactivations called “micro-arousals”. It is believed that SB is closely related to the central nervous system (both sympathetic and parasympathetic) [20,21,22,23,24].

Apart from the above-mentioned factors, other biological causes, e.g., DNA hypomethylation and genetic polymorphisms in the serotonergic system as aetiological factors for bruxism, still need further research [25,26]. Furthermore, the role of psychological factors in sleep bruxism has been investigated. A positive correlation has been observed between the prevalence of bruxism and raised stress-related catecholamine levels [27,28], anxiety [29,30], and a distressed type of personality [17,31,32,33]. Neuroticism has also been proven to increase muscle strength [34]. External factors, e.g., medications, drugs, alcohol, nicotine, tobacco, along with co-existing conditions, such as sexsomnia, restless legs syndrome, and obstructive sleep apnoea [33,35,36,37,38,39], have also been researched. Smoking and nicotine dependency in particular have been associated with a higher risk of sleep bruxism, with smokers being two to three times more likely to be bruxers [40,41,42]. It has been suggested that the explanation lies in nicotine accumulation in the body during awake state and then its gradual decrease during sleep. Seeing as nicotine induces acetylcholine, glutamate synaptic transmission, and increases the release of dopamine, it could, therefore, have an exciting effect on sleep bruxism [43].

Questions have also been raised about the possible hereditary aspect of bruxism [44]. Even though the aforementioned research, which was mainly evidence-based, cross-sectional, and clinical, provided valuable insight into bruxism aetiology, it also showed how multifaceted it was. Numerous possible above-mentioned factors need to be taken under consideration when trying to give a hypothesis on bruxism aetiology. This factor makes it difficult to design clinical studies. Accurate equipment is sometimes necessary to support those studies, which can in turn be expensive and difficult to obtain.

### 1.3. Assessment of Bruxism

A qualified and thorough assessment of bruxism is crucial for the further diagnosis and proper management of the condition. Severe bruxism can lead to tooth surface loss, fracture of dental restorations or teeth, hypersensitive or painful teeth, and loss of dental implants and periodontal support [45,46,47]. Despite its negative consequences, sleep bruxism has also been said to have positive aspects. It might have positive physiological capacities, for example, allowing an unobstructed airflow and helping with salivation during rest, therefore securing the wellbeing of the upper gastrointestinal tract [48].

Over the years, apart from explaining its multifaceted aetiology, a simple yet reliable diagnostic method of bruxism has also remained elusive. Although self-reports and clinical examination results are of interest when assessing sleep bruxism, their findings can be limited. To this day, polysomnography (PSG) with audio-video (AV) recordings has remained in the most high regard and are called a “gold standard” in bruxism diagnostics, even with their shortcomings pertaining to cost and the need for a suitable sleep laboratory [49,50,51].

According to the International Classification of Sleep Disorders (ICSD-3) the “clinical criteria” for the classification of SB are as follows: (A) the presence of regular or frequent teeth-grinding sounds occurring during sleep; (B) the presence of one or more of the following clinical signs: (1) abnormal tooth wear consistent with the above reports of tooth grinding during sleep; (2) transient morning jaw muscle pain or fatigue, temporal headache, and/or jaw locking upon awakening consistent with the above reports of tooth grinding during sleep [5,6].

The International Consensus of 2018 [8], proposed a grading system for bruxism to determine whether a certain assessment method of bruxism actually offers a credible outcome:

1. Possible sleep/awake bruxism is based on a positive self-report only.
2. Probable sleep/awake bruxism is based on a positive clinical inspection, with or without a positive self-report.
3. Definite sleep/awake bruxism is based on a positive instrumental assessment, with or without a positive self-report and/or a positive clinical inspection.

In recent years, due to growing awareness of bruxism among doctors and a rising need for efficient and reliable diagnostic methods for bruxism, portable devices measuring the electromyography (EMG) of masticatory muscles have been introduced. Monitoring jaw muscles’ activity and heart rates and offering the possibility of performing the examination overnight by the patient at their own home at a lower cost and with promising research results, portable EMG devices (e.g., Bruxoff, Bioelettronica, Turin, Italy) offer a useful solution for screening or possibly diagnosing patients with sleep bruxism [52,53,54]. This study used self-reports from patients, clinical inspection, and an instrumental assessment using a Bruxoff device. Therefore, it matched grade 3 of the above-mentioned grading system established by the International Consensus of 2018 [8].

The aim of this paper was to evaluate the accuracy of the Bruxoff device in screening and/or diagnosing patients with sleep bruxism. The null hypothesis of this paper was that the Bruxoff device is both a reliable screening and diagnostic method for SB.

## 2. Materials and Methods

### 2.1. Study Design

This study included 60 adult patients according to criteria listed in Table 1. For the study group, 35 patients qualified with respectively 25 patients for the control group. Patients were enrolled between June 2017 and March 2019. The study took place in the Laboratory of Masticatory Dysfunctions, Department of General Dentistry, Medical University of Lodz, Poland.

All patients were examined by the same trained dentist. No patient had missing incisors or cuspids and all missing teeth were premolars or molars. In the study group, 22 patients had full dentition (excluding third molars), 5 patients had 1 missing tooth, 4 patients had 2 missing teeth, 3 patients had 3 missing teeth, and 1 patient had 4 missing teeth, one per each quadrant. Subsequently, in the control group, 11 patients had full dentition (excluding third molars), 6 patients had 1 missing tooth, 2 patients had 2 missing teeth, 1 patient had 3 missing teeth (one in upper left quadrant and two in lower right quadrant), and 5 patients had 4 missing teeth (2 patients in first and second quadrants, two patients in third and fourth quadrants and one patient in first and fourth quadrant). The clinical examination focused on checking bruxism symptoms in the oral cavity, i.e., masticatory muscle pain, indentations on the tongue or lip and/or linea alba on the inner cheek, damage to the dental hard tissues, or mechanical wear of the teeth [8]. The Tooth-Wear Index (TWI) by Smith and Knight [56] (Table 2) was used to evaluate incisal and occlusal wear on a scale from 0 to 4.

Masseter muscles and temporal muscles on both sides were examined with an intraoral and extraoral palpation test. Both the origins and the heads of the muscles were tested for any presence of pain upon examination.

The study used a portable electromyography/electrocardiogram (EMG/ECG) device (Bruxoff, Bioelettronica, Turin, Italy) to confirm a bruxism diagnosis. Bruxoff consists of two concentric EMG electrodes, three ECG electrodes, and a small holter. The EMG electrodes monitor and bilaterally register masseter muscle activity, while the ECG electrodes record heart rate. All of the Bruxoff electrodes are disposable and suitable for one use only. Each examination with the Bruxoff device was performed by the patient during the course of one night, during sleep. The dentist explained in detail to each patient how to apply and use the Bruxoff device and then verified the patient’s understanding of the given instructions. Upon going to sleep, the patients were to apply two EMG electrodes on their masseter muscle and three ECG electrodes on their chest. The placement of the electrodes was explained in detail. After applying the electrodes and turning on the Bruxoff device, they were instructed to perform three maximum voluntary clenchings (MVCs) lasting 3 s each and separated by 10 s of rest at the beginning of the examination. Then, they were instructed to go to sleep. After waking up, they were told to switch off the device, remove the electrodes, and deliver the device back to the dentist. The greatest of the MVC measures was used for normalizing the EMG values as a percent of MVC. The collected data were analysed by the dedicated software, which was able to classify an SB episode, if the EMG burst was greater than 10% MVC and if it immediately followed (1–5 s interval) a heart rate increase of 20% with respect to the baseline [53].

Bruxoff was used to measure data consisting of the bruxism index (number of bruxism episodes per hour), mean heart rate, number of masseter muscle contractions (including tonic, phasic and mixed), and examination length (time between switching the device on and off). The data were collected, registered, and evaluated by the same trained dentist. Bruxism events were classified as either tonic (sustained EMG burst lasting more than 2 s), phasic (3 or more rhythmic EMG bursts of 0.25 to 2 s in duration), or mixed (both sustained and rhythmic). Sleep length could not be collected due to the lack of EEG electrodes.

Since the Bruxoff device does not have a built-in audio and/or video recording monitor, the cut-off criteria for bruxism were based on a publication by Carra et al. [57], pertaining to the diagnostic accuracy of sleep bruxism scoring in the absence of audio-video recording. The criteria were that subjects experiencing more than 2 SB episodes per hour of sleep received a diagnosis of sleep bruxism.

The study was approved by the Local Ethical Committee (RNN/173/17/KE). All patients signed the consent form prior the inclusion to the experiment.

### 2.2. Statistical Analysis

For qualitative variables, the structure indices were calculated and expressed in %. The following characteristics were calculated for measurable variables: arithmetic mean (x) and median (Me), as average values and standard deviation (SD), and the coefficient of variation (CV), as a measure of dispersion. The test value (z) and minimum (min) and maximum (max) values were also given. Before comparison of the averages, the normality of the quantitative variable distributions was checked using the Shapiro-Wilk test. Due to the distributions of variables significantly differing from the normal distribution, the non-parametric Mann-Whitney test was used to compare means in two groups (study and control). To compare the prevalence of muscle pain, bruxism symptoms in the study and the control group, the chi-square test of independence was used. In the study of the relationship between measurable variables, the rank correlation coefficient was calculated.

Statistically significant differences were found between average values or frequencies and those relations between variables for which the calculated test value was equal to or greater than the critical value read from the respective tables with the right number of degrees of freedom and probability of error *p* < 0.05. Sensitivity and specificity were calculated for the Bruxoff device.

## 3. Results

Patients from the study group were aged 20 to 56 years, the average age was 29.9 ± 8.35 years, and half of the respondents were under 28 years of age. Subjects in the control group were aged 22 to 66 years, the average was 35.0 ± 10.9 years, and half of the respondents had already completed 34 years. Comparison of the mean age in the study and the control groups showed a statistically significant difference (*p* < 0.05). It turned out that the patients from the study group were significantly younger than in the control group.

In terms of TWI, masticatory muscle pain, and other bruxism symptoms in the oral cavity, all patients within the study group showed signs of tooth wear and muscle pain along with other symptoms. The majority of patients (30 out of 35 subjects) in the study group presented a score 1 of TWI, whereas the other five subjects of the study group presented a score 2 of TWI.

There was no statistically significant difference in the length of the examination between patients in the study group and control subjects (*p* > 0.05). In the study group, the average time between switching the device on and off was 7 h 2 min ± 1 h 8 min, and in the control group, it was 7 h 11 min ± 1 h 18 min.

**Figure 1:**
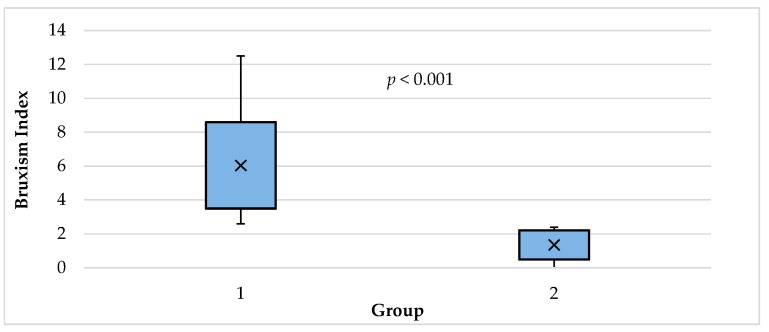
Bruxism index in the study group (1) and control group (2).

As expected, the comparison of the mean of the bruxism index in the study and the control group showed a statistically significant difference (*p* < 0.001). It turned out that patients from the study group had a significantly larger mean bruxism index than those in the control group: 6.04 ± 2.55 vs. 1.35 ± 0.86 (Table 3, Figure 1).

Women dominated in each group. The respective percentages were: 65.7% and 80.0%. In the study group, the bruxism index in women yielded values from 2.60 to 12.5, the average was 5.90 ± 2.74, and in half of the respondents, it did not exceed 5.5 (Table 4). In contrast, in men of the study group, the bruxism index ranged from 2.70 to 10.3, the mean was 6.30 ± 2.24, and in the half of respondents, it exceeded 5.95. The comparison of the bruxism index average values in the study group for men and women showed no statistically significant difference (*p* > 0.05).

In the control group, the bruxism index in the women assumed values from 0.0 to 2.4 (the average was 1.27 ± 0.94), and in men of the control group, the bruxism index ranged from 1.2 to 2.1 (the average was 1.68 ± 0.37).

Comparison of the mean of the bruxism index in the control group for men and women did not show a statistically significant difference (*p* > 0.05).

There were no statistically significant correlations between the bruxism index and the sleep length of patients (*p* > 0.05) in both the study and control groups, and in this case, the Spearman’s rank correlation coefficient was even closer to zero.

Comparison of the mean heart rate in the study and control groups showed a statistically significant difference (*p* < 0.05). It turned out that patients from the study group had an average heart rate lower than those in the control group: 59.6 ± 6.80 vs. 63.5 ± 4.86 beats per minute (Table 5).

A comparison of the mean number of all contractions of masseter muscle in the study and in the control group did not show a statistically significant difference in this respect (*p* > 0.05) (Table 6).

Further, the comparison of the mean number of phase contractions in the study and in the control group did not show a statistically significant difference in this range (*p* > 0.05) (Table 7).

In this case, however, it is worth noting that the average number of phase contractions (RMMA) in the study and the control group was very similar, amounting to 23.5 ± 13.2 and 24.0 ± 22.1, respectively.

There was also no statistically significant difference between the analysed groups compared to the average number of tonic contractions in the traces of this study (*p* > 0.05) (Table 8).

The averages in the study and the control group turned out to be almost the same: 41.6 ± 52.5 and 41.7 ± 37.4. What draws attention is that there was a very large variation of this variable, especially in the study group. The coefficients of variation were, respectively, 126.0% and 89.8%. A comparison of the mean number of mixed contractions in the study and in the control group did not show a statistically significant difference (*p* > 0.05) (Table 9). The respective averages were: 4.06 ± 4.96 and 4.40 ± 6.14. It should be noted that there was a very large variation of this variable, both in the study and control groups. The coefficients of variation were, respectively, 122.2% and 139.5%. As can be seen, the volatility of the number of mixed contractions in both groups exceeded 100.0%.

There were no statistically significant correlations between the bruxism index and the total number of masseter muscle contractions in both the study group and the control group, and the correlation coefficients did not differ from zero in a statistically significant manner (*p* > 0.05).

There were also no statistically significant correlations between the bruxism index and the number of phase and mixed contractions, both in the study group and in the control group (Table 10). Spearman’s rank correlation coefficients did not differ from zero in a statistically significant manner (*p* > 0.05). However, a statistically significant relationship was found between the level of bruxism and the number of tonic contractions in the study group (*p* < 0.05). It turned out that the higher the bruxism index, the more patients had tonic contractions. This relationship was moderately strong (Spearman’s rank correlation coefficient is 0.377, *p* < 0.05). In the control group, however, no such relationship was found. In this case, the Spearman’s rank correlation coefficient was very close to zero.

In relation to the cut-off criteria for bruxism (Table 11), data recorded with the Bruxoff device indicated that all subjects in the study group matched the criteria. Surprisingly, some subjects in the control group matched the criteria for bruxism, as well.

The sensitivity and specificity of Bruxoff were calculated (Table 12). The sensitivity of the test is the ratio of true positive results to the sum of true positives and false negatives. The obtained sensitivity of 100% means that all subjects suffering from bruxism would be recognized using the Bruxoff device. The specificity of the test is the ratio of true negative results to the sum of true negative and false positives. The estimated specificity of the device would mean that 76% of all healthy people in the diagnostic test will be marked as healthy.

## 4. Discussion

Thorough masticatory muscle examination and observation of bruxism symptoms in the oral cavity underpin the bruxism diagnosis. Like mentioned before, every patient qualified for the study group had muscle pain and bruxism symptoms in the oral cavity upon examination, along with a positive self-report of bruxism. Subsequently, patients who qualified for the control group presented no muscle pain upon examination, no bruxism symptoms in the oral cavity, and a negative self-report for bruxism. As expected, the bruxism index (the amount of bruxism episodes per hour) was significantly higher for the study group. All patients with muscle pain and/or bruxism symptoms turned out to be bruxers. Interestingly, some patients without masticatory muscle pain and/or bruxism symptoms also turned out to be bruxers, as assessed by the Bruxoff device (Table 12). This result could be subject to number of interpretations/explanations. It could be due to the positive and regulatory aspect of sleep bruxism in terms of providing an unobstructed airway for breathing in obstructive sleep apnea (OSA) and regulating salivation during sleep, as mentioned before. Therefore, patients showing no symptoms of sleep bruxism could still clench their teeth during some nights, but not to a degree that would cause them to show symptoms of sleep bruxism. Patients were not tested for OSA due to the limitations of the device. Nevertheless, this finding suggests that a bruxism diagnosis based solely on both EMG and ECG analysis could still overestimate SB [58].

In terms of the accuracy of the Bruxoff device tested in this study, while the device turned out to have sensitivity of 100%, its specificity of 76% still leaves room for improvement, as there is a chance of providing a healthy subject with a false positive bruxism diagnosis. However, for this study to have a more thorough assessment of these parameters, the number of patients in both the study and the control group would have to be much larger. The producers of Bruxoff valued its sensitivity at 92% and its specificity at 85%. Other studies also showed the high sensitivity and specificity of the Bruxoff portable device (92.3% and 91.6%, respectively), as well as a high correlation and high agreement between Bruxoff and the PSG readings [52,58].

Moreover, the sensitivity and specificity outcome obtained in this study could suggest that the three maximum voluntary contractions (MVC) at the beginning of each examination were not performed by the patients with actual maximum strength. Therefore, this could mean that an examination performed while the patient is not watched over by a specialist could be limited, since there is no possibility of monitoring how the patient performs the examination with Bruxoff. According to The International Consensus of 2018 [8], the cut-off points for establishing the presence or absence of bruxism should not be used in otherwise healthy individuals. Rather, bruxism-related masticatory muscle activities should be assessed in the behaviour’s continuum. Even though new research shows that bruxism could be connected with musculoskeletal pain, it does not support a direct and straight connection between them, but rather suggests a complex approach taking into account the presence of other risk factors [59].

Research showed that bruxism affects women more commonly than men [3,60]. In this study, in terms of gender, no statistically significant difference between the study and the control groups was found. In each individual group, females dominated, and the male/female ratio in each group was not equal. However, this fact did not affect the mean bruxism index calculated separately for the men and women within each group. The limited number of participants was taken into account, while calculating all statistical differences, and, therefore, only highly visible statistical differences were considered significant (*p* < 0.05). These findings are consistent with other papers that report no sex differences in terms of sleep bruxism [1,44,61,62,63]. Moreover, the prevalence of women observed in studies on the subject results from their higher awareness and eagerness to seek professional help [2].

In terms of the number and types of muscle contractions (tonic, phasic, and mixed) the results showed no significant differences between the study and the control group. A previous study showed the absence of a significant correlation between the number of masseter contraction per hour and the number of SB episodes per hour [53]. In terms of a correlation between a specific type of contraction and the bruxism index, there was a surprisingly moderate correlation between the tonic (clenching) contractions and the bruxism index. The more tonic contractions there were, the higher the bruxism index. According to some literature, a higher amount of tonic activity could be associated with bruxism while awake and morning muscle symptoms [45,64,65]. Therefore, it could be said that the morning muscle pain and fatigue reported by all patients within the study group were caused by clenching-type (tonic) sleep bruxism [66]. However, other heavily supported research [67,68,69,70,71] showed an increased number of phasic contractions in patients with sleep bruxism, which was not found in this study. This could suggest that the reliability of the Bruxoff device in this particular spectrum needs further study.

Surprisingly, the study group has showed a lower mean heart rate compared to the control group, even though episodes of sleep bruxism are said to be characterised by short tachycardic outbursts. This could be explained by the trigemino-cardiac response (TCR) that was triggered by episodes of sleep bruxism and which has been shown to cause bradycardia [72,73]. That, in turn, might have resulted in lower average heart rates in subjects from the study group. However, this finding could be also due to the fact that the ECG capabilities of Bruxoff are limited, since the detected heart rate signal was used only to identify the RMMA. As the producers of Bruxoff state, the device has not been designed and engineered for electrocardiographic investigations and is not suitable for diagnosing cardiovascular diseases. Moreover, such an outcome could have been produced by the limitations of the study, as the patients were not supervised when recording the data.

In terms of screening use, Bruxoff showed significant potential thanks to its EMG and ECG electrodes, which monitor both masseter muscle activity and the heart rate. A bruxism diagnosis based only on the surface EMG analysis tends to overestimate SB [53]. These two parameters are, as previously mentioned in this paper, the foundations of the most valid theories pertaining to bruxism aetiology. Previous papers have also confirmed Bruxoff’s potential as a screening device for patients with sleep bruxism [53,54,74]. In light of this, the authors suggested that the use of such portable EMG devices, e.g., Bruxoff (Bioelettronica, Italy), BiteStrip (Alldent, Australia), and GrindCare (Sunstar, Switzerland), could be considered by clinicians dealing with bruxism and could deliver promising results [52,53,54,75,76,77]. Further, devices measuring grinding, e.g., Bruxchecker (Scheu-Dental, Germany), should be taken into consideration [78]. Perhaps these portable devices could someday challenge the “golden standard” that is PSG with audio and visual recording. However, even with the research results positioning portable EMG devices among the most reliable and easy to use methods for diagnosing bruxism, the complexity, sensitivity, and irreplaceable value of audio-video recordings in PSG still places it as the most detailed and valid diagnostic method for sleep bruxism. Furthermore, there have been developments in the form of portable sleep monitoring devices, e.g., the Nox-T3 Portable Sleep Monitor (Nox Medical, Reykjavik, Iceland), which seem to answer the need for simplified, yet detailed, diagnostic equipment in terms of sleep bruxism and sleep medicine in general [6,70].

The results of this study also showed a significant age difference between the groups, with the study group being much younger than the control group. Upon examining dozens of prospective participants, it became clear that, consistent with the epidemiologic data, sleep bruxism decreases with age—an occurrence that has been linked with coping mechanisms and responses to stress [3,79].

The Bruxoff study possesses several limitations that should be taken into account, like the large range of ages between the study and the control group. Further, due to the fact that Bruxoff does not have audio-video recording, it cannot register either sound or visual activity (e.g., grinding). Moreover, patients’ activities during sleep could possibly influence the Bruxoff readings. Due to the lack of EEG monitoring, the device cannot truly register patients’ sleep lengths, as there is no possibility of knowing, when the patient actually falls asleep and wakes up. The fact that the examination was performed for one night only might be an issue as the repeatability of the results could not be evaluated. Unfortunately, these two elements (audio–video and EEG) are crucial for a comprehensive and accurate diagnosis of sleep bruxism. Therefore, in spite of having EMG and ECG electrodes, Bruxoff cannot be considered a reliable match for polysomnography.

Bruxoff could be a useful device for dentists, who do not specialise in the field of temporomandibular disorders and whose experience with the clinical signs and symptoms of bruxism is limited. Furthermore, Bruxoff could be useful for doctors who conduct prosthodontic and periodontal treatments and are in need of an accurate method for screening and/or diagnosing their patients for bruxism.

## 5. Conclusions

Within the limitations of the present study, it can be concluded that Bruxoff is a simple screening device that can be safely used to evaluate masseter muscle activity during sleep. Since the device does not have a built-in microphone and/or video camera and, therefore, cannot record grinding sounds, the objective capabilities of Bruxoff as a single diagnostic device for sleep bruxism are limited.

## Figures and Tables

**Table 1:** Inclusion and exclusion criteria for the study group and the control group.

	Inclusion Criteria	Exclusion Criteria
Study group	- willing to participate in the study - between 18 and 66 years old - male or female - in general good health - present clinical bruxism symptoms in the oral cavity including masticatory muscle pain, indentations on the tongue or lip and/or linea alba on the inner cheek, damage to the dental hard tissues or mechanical wear of the teeth [8] - self-reported bruxism	- unwilling to participate in the study - under 18 years old - severe mental illness - severe neuromuscular illness - during prosthodontic rehabilitation - more than two missing teeth per quadrant (excluding third molars) [55] - under medication, which impairs neuromuscular system activity - under anti-psychotic medication - any severe chronic illness - cognitive disability - active inflammation - present oncologic condition - smoking
Control group	- willing to participate in the study - between 18 and 66 years old - male or female - in generally good health - no bruxism symptoms present including muscle pain - no self-reported bruxism	

**Table 2:** Tooth Wear Index (TWI) according to Smith and Knight.

TWI	Tooth Surface	Criterion
**0**	Buccal/Lingual/Occlusal/Incisal Cervical	No loss of enamel surface characteristics. No loss of crown contours.
**1**	Buccal/Lingual/Occlusal/Incisal Cervical	Loss of enamel surface characteristics. Minimal loss of crown contours.
**2**	Buccal/Lingual/Occlusal	Loss of enamel exposing dentine for less than one third of examined surface.
	Incisal	Loss of enamel just exposing dentine.
	Cervical	Defect less than 1 mm deep.
**3**	Buccal/Lingual/Occlusal	Loss of enamel exposing dentine for more than one third of examined surface.
	Incisal	Loss of enamel and substantial loss of dentine.
	Cervical	Defect 1–2 mm deep.
**4**	Buccal/Lingual/Occlusal	Complete enamel loss or secondary dentin exposure or pulp exposure.
	Incisal	Exposure of pulp or secondary dentine.
	Cervical	Defect more than 2 mm deep or pulp exposure or secondary dentine exposure.

**Table 3:** Comparison of the average values of the bruxism index in patients from the study group and in the control group.

Group	Calculated Parameters of the Bruxism Index					
	min	max	x	Me	SD	CV (%)
**Study**	2.60	12.50	6.04	5.50	2.55	42.30
**Control**	0.00	2.40	1.35	1.70	0.86	63.80
Comparison	**z = 6.552; *p* = 0.0000**					

x, arithmetic mean; Me, median; SD, standard deviation; CV, coefficient of variation; min, minimum; max, and maximum.

**Table 4:** Comparison between gender and the average value of the bruxism index in patients from the study and the control groups.

	Number of Women	Mean Bruxism Index ± SD	Number of Men	Mean Bruxism Index ± SD
**Study group**	23	5.90 ± 2.74	12	6.30 ± 2.24
**Control group**	20	1.27 ± 0.94	5	1.68 ± 0.37

**Table 5:** Comparison of mean heart rate in patients in the study group and in the control group.

Group	Calculated Heart Rate Parameters (Number of Beats/min)					
	**min**	**max**	**x**	**Me**	**SD**	**CV (%)**
**Study**	45.00	72.00	59.60	60.00	6.80	11.40
**Control**	52.00	73.00	63.50	62.00	4.86	7.70
Comparison	**z = 2.272; *p* = 0.0231**					

**Table 6:** Comparison of mean masseter muscle contractions in patients in the study group and in the control group.

Group	Calculated Parameters of Overall Number of Masseter Contractions					
	**min**	**max**	**x**	**Me**	**SD**	**CV (%)**
**Study**	17.0	452.0	102.0	93.0	75.4	73.9
**Control**	15.0	346.0	131.0	115.0	91.6	69.6
Comparison	**z = 1.311; *p* = 0.190**					

**Table 7:** Comparison of mean phasic contractions in patients in the study group and in the control group.

Group	Calculated Parameters of the Number of Phasic Contractions					
	**min**	**max**	**x**	**Me**	**SD**	**CV (%)**
**Study**	3.0	49.0	23.5	23.0	13.2	56.0
**Control**	0.0	81.0	24.0	14.0	22.1	92.2
Comparison	**z = 0.607; *p* = 0.544**					

**Table 8:** Comparison of mean tonic contractions in patients in the study group and in the control group.

Group	Calculated Parameters of the Number of Tonic Contractions					
	**min**	**max**	**x**	**Me**	**SD**	**CV (%)**
**Study**	2.0	296.0	41.6	24.0	52.5	126.0
**Control**	0.0	178.0	41.7	30.0	37.4	89.8
Comparison	**z = 0.629; *p* = 0.529**					

**Table 9:** Comparison of mean mixed contractions in patients in the study group and in the control group.

Group	Calculated Parameters of the Number of Mixed Contractions					
	**Min**	**max**	**x**	**Me**	**SD**	**CV (%)**
**Study**	0.00	19.00	4.06	1.00	4.96	122.20
**Control**	0.00	23.00	4.40	2.00	6.14	139.50
Comparison	**z = 0.075; *p* = 0.940**					

**Table 10:** Correlation between each type of contraction and the bruxism index in the study group and in the control group.

Contraction	Study Group			Control Group		
	Rank Correlation	Test Value *t*	Significance *p*-Value	Rank Correlation	Test Value *t*	Significance *p*-Value
**Phasic**	0.289	1.732	0.093	-0.131	0.635	0.532
**Tonic**	0.377	2.339	0.0255	0.103	0.499	0.623
**Mixed**	0.123	0.713	0.481	0.141	0.685	0.500

**Table 11:** The cut-off criteria for bruxism evaluated in the study group and in the control group using the Bruxoff device.

Criteria	% of Subjects Matching the Criteria	
	Control	Study
more than 2 bruxism episodes per hour of sleep	24	100

**Table 12:** Comparison between the clinical examination result and Bruxoff result in diagnosing bruxism.

	Clinical Examination Result		
Bruxoff Result	Bruxism	No Bruxism	In Sum
**Bruxism**	35	6	41
**No bruxism**	0	19	19
**In sum**	35	25	60
	**Sensitivity: 100%**	**Specificity: 76%**	
